# The Vital Dye CDr10b Labels the Zebrafish Mid-Intestine and Lumen

**DOI:** 10.3390/molecules22030454

**Published:** 2017-03-13

**Authors:** Veronika Sander, Shantanu Patke, Jung Y. Lee, Young-Tae Chang, Alan J. Davidson

**Affiliations:** 1 Department of Molecular Medicine & Pathology, University of Auckland, Auckland 1142, New Zealand; v.sander@auckland.ac.nz (V.S.); spat261@aucklanduni.ac.nz (S.P.); 2 Department of Chemistry & NUS MedChem Program of Life Sciences Institute, National University of Singapore and Singapore Bioimaging Consortium, A* STAR, Singapore 138667, Singapore; lsilj@nus.edu.sg (J.Y.L.); chmcyt@nus.edu.sg (Y.-T.C.)

**Keywords:** zebrafish, gut development, mid-intestine, CDr10b, vital dye, laser ablation

## Abstract

We describe the use of the fluorescent reporter compound CDr10b to label mid-intestinal structures in zebrafish larvae after simple immersion. CDr10b is deposited into the gut where it initially fills the lumen and is excreted. Using laser-mediated injury of the intestine, we show that CDr10b provides a useful readout of the integrity and repair of the epithelial cell barrier. In addition, CDr10b specifically labels the absorptive mid-intestine segment that is analogous to the mammalian small intestine. By perturbing retinoic acid signaling, which regulates the size of the mid-intestine segment, we show that CDr10b is a valuable tool to rapidly assess developmental malformations of the intestine in live animals.

## 1. Introduction

The intestinal epithelium is comprised of a monolayer of columnar epithelial cells that form a barrier between the underlying tissue and the hostile environment of the gut lumen. Enterocytes are the prevalent cell type of the epithelium and play a key role in nutrient absorption. Intestinal development, homeostasis and disease have been well described in mammals [1]. The zebrafish represents a simple, yet relevant vertebrate model organism for studying intestinal development and function. Its size, rapid development, transparency (until early adulthood stages) and fecundity allow for fast, high-resolution imaging and high-throughput approaches in vivo that are cumbersome and expensive in rodent models. Moreover, zebrafish gene and organ function are well conserved with higher vertebrates [2,3,4].

**Figure 1:**
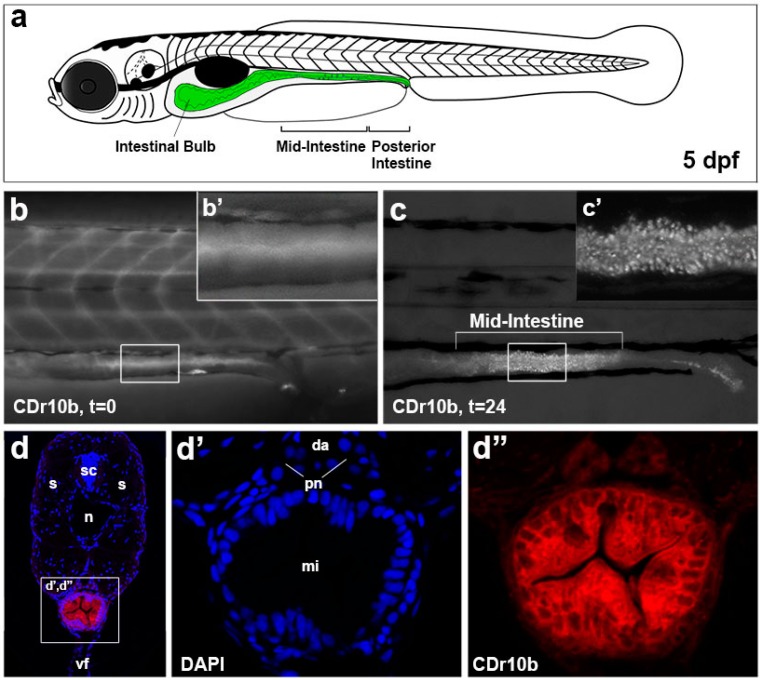
CDr10b labels the mid-intestine. (**a**) Schematic of a 5 dpf larva with the gut in green; (**b**,**b’**) After treatment (100 nM, 45 min), CDr10b is found in the blood and gut lumen (t = 0); (**c**) 24 h post treatment (t = 24), cells of the mid-intestine are stained by CDr10b; (**c’**) Localization of CDr10b in a punctate-pattern in enterocytes; (**d**–**d’’**) Transverse section through the mid-intestinal segment showing CDr10b staining (red in **d**) and nuclear DAPI staining (blue in **d**). da, dorsal aorta; dpf, days post fertilization; mi, mid-intestine; n, notochord; pn, pronephros; t, time post treatment; s, somites; sc, spinal cord; vf, ventral fin.

Zebrafish gut development starts at approximately 20 h post fertilization (hpf) with endodermal precursors forming the primitive gut, a thin rod-like cell layer extending from the future mouth to the future anus of the embryo. The primitive gut cells then polarize to become a columnar epithelium, and junctional complexes are formed between the cells, a prerequisite to lumen inflation and establishment of the epithelial barrier. These processes are accompanied by proliferation along the entire length of the intestinal tube. Around 72 hpf, proliferation declines and the intestinal epithelial cells undergo differentiation into the three lineages of the mature gut epithelium: absorptive enterocytes, mucus-producing goblet cells and hormone-secreting enteroendocrine cells. Gut development is completed by 5 days post fertilization (dpf), when the yolk is fully absorbed and the larva begins to feed and digest. The gut tube is then subdivided into three segments: the intestinal bulb in the most rostral region, characterized by an expanded lumen and epithelial folding, is primarily comprised of enterocytes and enteroendocrine cells; the mid-intestine is demarcated by the presence of goblet cells and enterocytes with large, supranuclear vacuoles; and the posterior-intestine is devoid of endocrine and goblet cells [5,6] (Figure 1a). Gene expression analyses of the individual segments of the adult zebrafish intestine have demonstrated a high similarity of the intestinal bulb and mid-intestine to the mammalian small intestine, while the posterior fish intestine mostly resembles the mammalian colon [3].

Intestinal development is regulated by a combination of growth factor gradients, including Wnt (Wingless-Type MMTV Integration Site), Fgf (Fibroblast growth factor) and Bmp (Bone morphogenetic protein) at the posterior end of the endoderm. In addition, retinoic acid (RA) signaling plays a dose dependent role in patterning of the anterior–posterior (A–P) body axis including the endoderm [7]. In zebrafish, lowering RA production strongly affects the formation of the pancreas, liver and intestine [8,9]. Under healthy conditions, the intestinal barrier prevents a multitude of potentially harmful factors such as pathogens, pharmaceuticals, bile and toxins from entering the body. For minor intestinal irritations, a rapid and efficient repair process called “restitution repair” occurs and involves the collective migration of non-injured intestinal epithelial cells to close the wound, restoration of the junctional complexes and proliferation to recover intestinal homeostasis [10]. Recurrent or excessive perforation of the intestinal epithelium, however, leads to a breakdown of its integrity, which in turn causes chronic inflammation conditions including different forms of intestinal bowel disease (IBD) [11]. With a significant clinical need for new treatments and a better understanding of the molecular processes underlying intestinal repair, zebrafish has proven to be a useful model for recapitulating IBD [12,13].

In vivo visualization of the gut tube, its motility and intestinal cell types is essential to study intestinal development and disease. In zebrafish, various techniques have been described, including microgavage of fluorescently-labeled dextrans and microspheres, which allows for precise temporal and spatial regulation of dye delivery [14,15]. A readout for organs involved in fatty acid metabolism including the intestine, pancreas and liver is achieved by feeding BODIPY-tagged fatty acid chains that, depending on the chain length, label different organs and cell types [16]. A drawback of these techniques is their laborious way of administration (gavage, and preparation of a fresh egg yolk-containing emulsion for BODIPY analogs).

In addition, small fluorescent compounds have proven useful tools for imaging tissues and subcellular structures, in particular in zebrafish, as their external development allows testing of small chemicals by simply adding them to the embryo water. In previous work, we have developed a diversity oriented fluorescence library (DOFL), and used it to screen for specific labeling of structures in larval zebrafish [17,18]. Here, we demonstrate that the DOFL molecule CDr10b, originally described as a live cell marker of microglia in mice [19], fills the gut lumen during larval intestinal development and, upon completion of development, specifically labels absorptive cells in the mid-intestinal segment from 5 dpf onwards.

## 2. Results and Discussion

### 2.1. CDr10b Labels the Larval Mid-Intestine

From a screen of DOFL molecules in zebrafish larvae, we identified CDr10b to be taken up by 5 dpf zebrafish from the fish water (100 nM, 45 min), possibly by direct swallowing and/or via the circulation as the compound is initially detected in the blood. CDr10b accumulates in the intestinal bulb and passes through the gut by peristalsis (Figure 1b,b’). Within 24 h, CDr10b in the intestinal bulb is cleared and the intestinal epithelial cells of the mid-intestine remain labeled with CDr10b in a punctate pattern (Figure 1c,c’). This staining corresponds to the previously described Neutral Red-positive region (Figure S1) [13]. CDr10b persists after fixation in 4% paraformaldehyde for at least three months (data not shown), allowing histological analysis of the preserved tissue. Transverse sections on the level of the mid-intestine revealed a cytoplasmic localization of CDr10b (Figure 1d–d’’). Sagittal sections clearly show that CDr10b is taken up by all cells in the mid-intestinal epithelium. This observation suggests an uptake mechanism specific for the mid-intestinal segment. Extended laser exposure to tissue sections revealed that CDr10b exhibits high photostability (Figure S2). In contrast to microglia, where CDr10b labels cytoplasmic structures by covalent binding, the non-chloroacetylated version (CDr10a [19]) remained stably bound after washout and 3-day incubation to mid-intestinal cells in our larval zebrafish model, suggesting a non-covalent mechanism of labeling (Figure S3). Thus, the thiol-reactive chloroacetyl motif does not seem necessary for labeling of the zebrafish gut.

**Figure 2:**
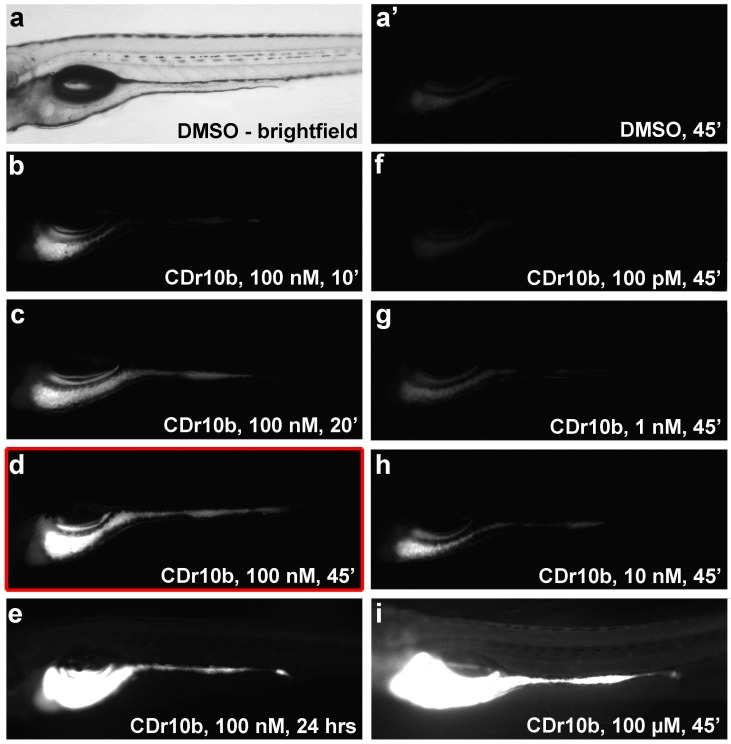
Dosage and duration of CDr10b treatment. Larvae were treated at 5 dpf with 0.01% DMSO or CDr10b at the doses and duration indicated in the panels, and photographed 24 h post treatment (6 dpf). (**a**) Brightfield image of a 5 dpf control (DMSO-treated) larva. (**a’**) Same specimen photographed using a red filter set. Only background fluorescence is detectable; (**b**–**e**) Increasing incubation times with constant dosage (100 nM CDr10b); (**f**–**i**) Increasing dosage of CDr10b with constant incubation time (45 min). All images were captured using the same exposure settings. Red box: Optimal conditions for CDr10b staining. For survival rates see Table S1. ’, minutes; hrs, hours; t, time post treatment.

Toxicity of CDr10b was examined by treating larval zebrafish with increasing doses over different incubation times. CDr10b was found to be non-toxic even at a 1000× higher concentration (100 μM), with 24 h survival rates comparable to control larvae. Continuous immersion in CDr10b for 24 h did not increase toxicity but led to a higher intensity of fluorescence in the gut and persistence of CDr10b in the circulation. We also tested lower doses and shorter incubation times and observed a detection limit at 100 nM for 20 min or 10 nM for 45 min (Figure 2, Table S1). Taken together, the optimal conditions for robust mid-intestine specific labeling were determined to be 100 nM for 45 min (Figure 2d). Long-term survival of CDr10b-treated larvae up to 4 weeks was comparable to control (DMSO-treated) larvae (57%, *n* = 43/75 and 48%, *n* = 15/31, respectively).

**Figure 3:**
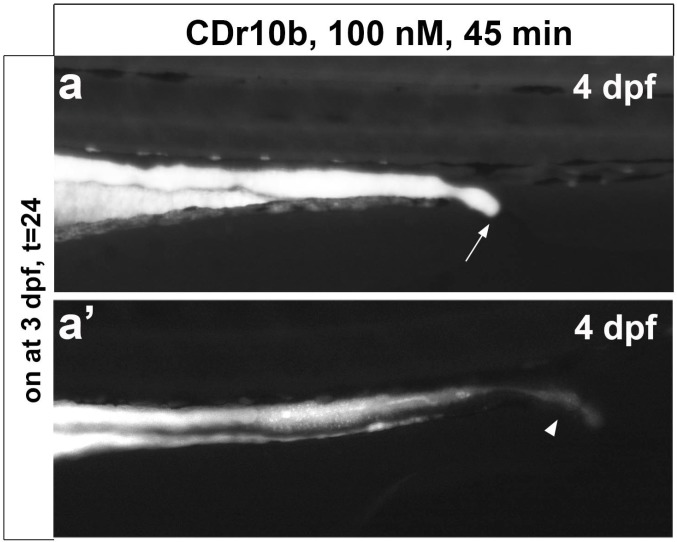

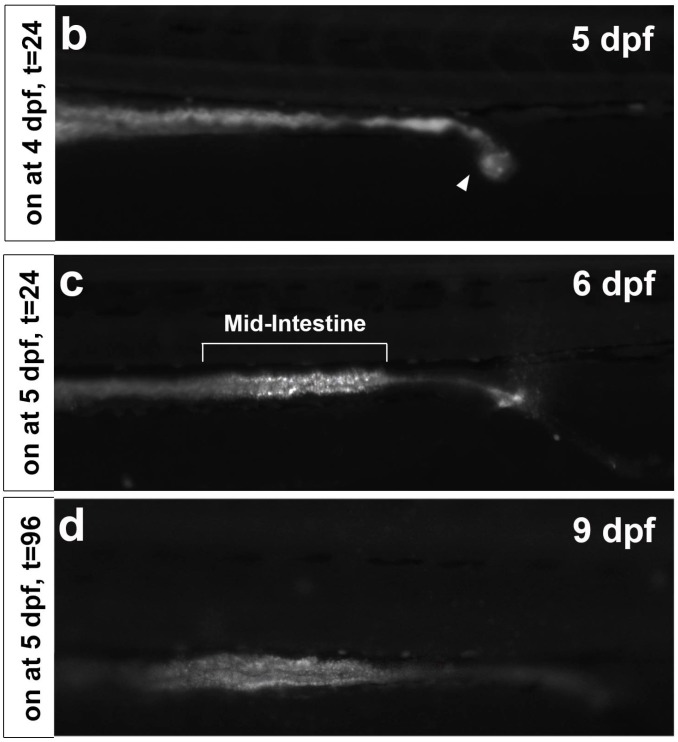
Stages of CDr10b staining. (**a**,**a’**) CDr10b taken up at 3 dpf stays in the intestinal lumen and can be used to visualize the timing of cloaca opening. Arrow in a, closed cloaca. (**b**) CDr10b treatment at 4 dpf results in luminal staining with excretion through the cloaca (arrowheads in **a’** and **b**); (**c**) CDr10b treatment at 5 dpf leads to uptake of the dye specifically by mid-intestinal epithelial cells; (**d**) The CDr10b signal in the mid-intestine is stable 4 days post treatment (t = 96). dpf, days post fertilization; t, time post treatment.

We next examined CDr10b staining over the course of development by exposing larvae of 3, 4 and 5 dpf to CDr10b. In concordance with the immature status of the gut at 4 dpf (a non-absorptive yet peristaltic tube), we found CDr10b only in the gut lumen with ~50% of the larvae displaying an open cloaca, consistent with fusion of the gut to the cloaca occurring at this stage [5]. Those animals with open passage to the cloaca showed decreased CDr10b intensity and the compound was actively excreted (Figure 3a,a’,b). Specific mid-intestine staining became obvious after 5 dpf (Figure 3c). This observation corresponds to the functional segmentation of the intestinal tube, which is largely complete by 5 dpf [5]. CDr10b is stable for at least 4 days post treatment (Figure 3d).

### 2.2. CDr10b Allows Detection of Intestinal Patterning Defects

**Figure 4:**
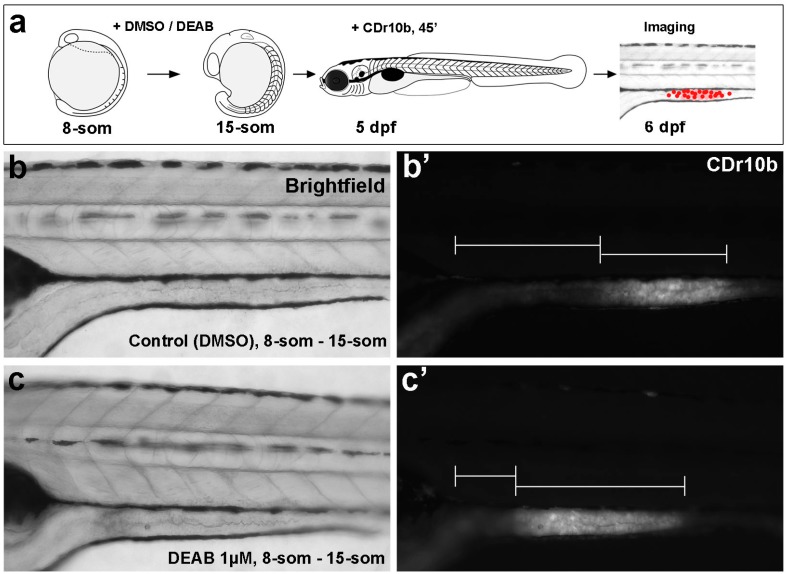
CDr10b labels developmental malformation of the intestine. (**a**) Outline of the experiment; (**b**,**c**) Brightfield images reveal that diethlyaminobenzaldehyde (DEAB) treatment during early development does not affect the morphology of the larvae; (**b’**,**c’**) Detection of the CDr10b signal reveals that the mid-intestinal segment is shifted towards anterior upon retinoic acid (RA) inhibition.

We next tested the applicability of CDr10b for detection of aberrant gut development. Altering the concentration of RA along the A–P axis of the embryo changes how mesodermally and endodermally derived tissues are patterned. However, the role of RA signaling in the formation of the mid- and posterior-intestine segments has not been examined [8,9,20,21]. To evaluate the role of RA on mid-intestinal patterning, we treated embryos with diethlyaminobenzaldehyde (DEAB), an inhibitor of the Aldehyde dehydrogenase 1 a2 enzyme that converts retinaldehyde to retinoic acid [22]. We determined that treating embryos from the 8-somite stage to 15-somite stage with 1 μM DEAB permitted survival to 5 dpf without severe toxicity (Figure 4a–c). Labeling these DEAB-treated animals with 100 nM CDr10b and then analyzing them at 6 dpf revealed a shift in the CDr10b-labeled mid-intestinal segment towards the anterior compared to control embryos (*n* = 27/28; Figure 4b’,c’; Table S2). As the overall length and morphology of the larvae was unaffected, this result supports the notion that RA plays a key morphogenic role in establishing the segmental patterning of the mid- and posterior-intestine. This assay demonstrates the use of CDr10b for fast detection of patterning defects in contrast to the traditional, but more time-consuming, approach of whole mount in situ hybridization for marker gene transcripts in fixed larvae.

### 2.3. CDr10b as a Readout for Intestinal Integrity

**Figure 5:**
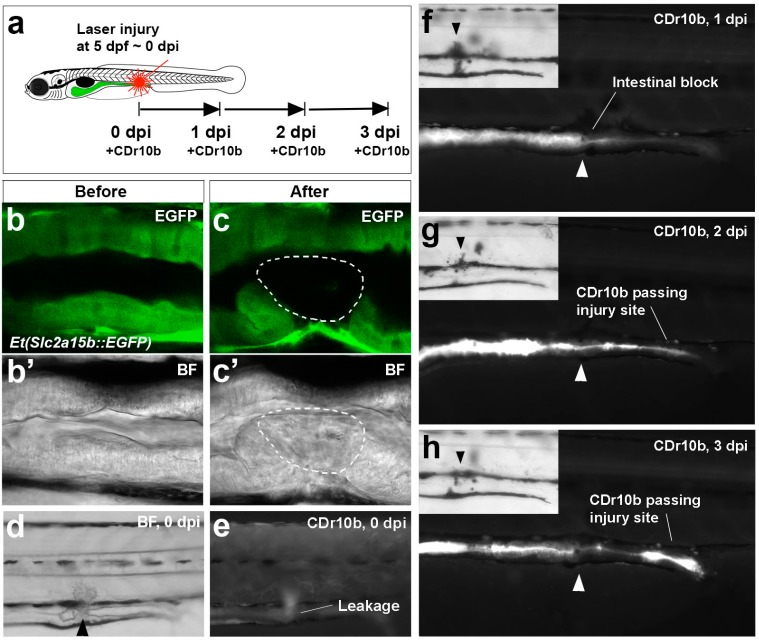
CDr10b as a readout for intestinal injury and regeneration. (**a**) Outline of the experiment; (**b**–**c’**) Confocal live imaging of mid-intestinal epithelium of a *Et(Slc2a15b::EGFP)* larva before and after laser cell ablation. Detection of the green fluorescence protein (GFP) signal (**b**,**c**) and the corresponding brightfield view (**b’**,**c’**). Intact epithelium (**b**,**b’**). After laser exposure, the cells have lost GFP expression and appear necrotic (white outline in **c** and **c’**); (**d**,**e**) Immediately after injury (0 dpi), the intestinal tube is partially blocked (brightfield in **d**) and CDr10b leaks into the surrounding tissue (CDr10b signal in **e**); (**f**–**h**) The same fish photographed at 1, 2 and 3 dpi after CDr10b treatment shows the flow of CDr10b blocked at 1 dpi, and regeneration with successful excretion of CDr10b by 3 dpi. Arrowheads mark the site of injury. BF, brightfield; dpi, day(s) post injury.

To address whether CDr10b is useful as a tool to examine the integrity of the intestinal epithelial cell barrier, we first tested it in the EDTA-gavage assay, which disrupts intestinal tight junctions and causes leakage of lumenal dyes into the interstitium and vascular space [15]. We observed increased levels of the dye in the circulation and lower levels remaining in the intestinal tube, consistent with leakage of CDr10b through the epithelium (Figure S4). Next, we developed a model of laser-induced small intestine injury and repair and tested CDr10b. Illumination of one side of the mid-intestine in 5 dpf larvae for 6 min with 405 nm laser light induced cell ablation of approximately 5–6 intestinal epithelial cells (Figure 5a–c′). For targeting the intestinal epithelium, the *Et(Slc2a15b::EGFP)* line generated from an in-house enhancer trap screen and expressing green fluorescence protein (GFP) in the gut (unpublished, see Section 3), was used. Initially, the laser-induced lesion is manifested as complete or partial blockage of the mid-intestinal tube, with CDr10b backing up and leaking into the surrounding tissue at the site of injury (*n* = 22/26; Figure 5d,e). Following the same larva over the course of 3 days with daily CDr10b treatment, we found evidence of mid-intestinal regeneration, with the blockage resolving and transit of CDr10b to the cloaca (Figure 5f–h). This novel assay highlights the use of CDr10b as a strategy to address the regenerative mechanisms governing intestinal repair, which most likely correspond to restitution repair seen in mammals [11].

## 3. Materials and Methods

### 3.1. Synthesis and Characterization of CDr10b

Details on synthesis and chemical characterization of CDr10b can be found in Leong et al. [19].

### 3.2. CDr10b/CDr10a

Lyophilized CDr10b and CDr10a were resuspended in dimethyl sulphoxide (DMSO) for a stock solution of 100 μM and stored at −20 °C.

### 3.3. Zebrafish Maintenance and Stocks

All zebrafish experiments were performed in accordance with the University of Auckland Animal Ethics Committee. Zebrafish (Tubingen wild type) were maintained at 28 °C under standard conditions. Embryos were raised in E3 medium [23]. *Et(Slc2a15b::EGFP)* was generated from an in-house enhancer trap screen in which a minimal promoter of 1132 bp from the Slc2a15 gene was used to drive GFP following Tol2-mediated insertion into the genome. The insertion site is unknown and will be characterized in detail elsewhere.

### 3.4. Zebrafish Treatment

Zebrafish larvae at 3, 4 or 5 dpf were incubated for 45 min in 1 mL of 100 nM CDr10b in E3 (unless stated otherwise) in 6-well plates at 28 °C. Larvae were subsequently washed twice with E3 and left to develop until imaging 24 h later. CDr10a was administered the same way. *N*,*N*-diethylaminobenzaldehyde (DEAB) treatment was performed by incubating embryos in 1 mL of 1 μM DEAB in E3 from 8-somite stage to 10- or 15-somite stage, at 28 °C. As control, 0.01% DMSO in E3 was used. After the treatment, the embryos were washed twice with E3 and left to develop until CDr10b staining at 5 dpf, and imaging at 6 dpf.

### 3.5. Imaging

Live embryos were imaged using a fluorescent compound microscope (Nikon Eclipse 80i, Nikon, Melville, NY, USA) equipped with a digital camera (Hamamatsu C4742-80-12AG). Embryos were anesthetized with 0.2 mg·mL^−1^ tricaine and placed in a 3 cm petri dish containing 1% agarose with larva-sized grooves for immobilization. Histological sections were imaged with a Zeiss LSM 710 inverted confocal microscope. Photostability was measured before and after 5 min of bleaching with a 488 nm laser.

### 3.6. Duration, Dose and Toxicity

The 5 dpf larvae were exposed to CDr10b concentrations ranging from 100 pM to 100 μM. Incubation times varied from 10 min to 24 h. A minimum of 15 larvae were tested per condition. The toxicity of CDr10b and CDr10a was determined by counting the number of surviving larvae at 24 h post treatment (24 h survival rate). CDr10b signals were analyzed 24, 48 and 72 h post treatment (Table S1). For assessment of long-term survival, larvae were raised in 3.5-liter tanks of Tecniplast’s ZebTec Zebrafish housing system. Surviving fish were counted at 28 dpf.

### 3.7. Histology

The 6 dpf larvae were fixed overnight in 4% Paraformaldehyde at 4 °C. After washing with PBS + 0.1% Tween, fixed larvae were transferred into an embedding mould, orientated and filled with embedding medium (1% low-melting agarose, 0.9% agar, 5% sucrose). Once solidified, the blocks were transferred into a 30% sucrose solution and incubated at 4 °C until saturated with sucrose (overnight), then removed from the solution and stored at −20 °C. The 12 μm sections were cut on a Leica cryostat (CM-3050-S), collected on Superfrost Plus slides (Fisher Scientific, Pittsburgh, PA, USA), dried at room temperature and stored at −20 °C. For imaging, sections were thawed at room temperature for 20 min, washed in PBS and mounted in ProLong Gold plus DAPI for nuclear staining.

### 3.8. Laser Ablation

Damage of the mid-intestinal epithelium was inflicted with 405 nm laser light induced cell ablation (photobleaching) of the ventral side of the intestinal epithelium for 6 min using a SIM scanner on a Olympus FV1000 confocal microscope (Olympus, Tokyo, Japan).

## 4. Conclusions

In summary, we describe a new use of the chemical compound CDr10b as a non-toxic vital dye that labels the zebrafish intestinal lumen and mid-intestine epithelium following simple immersion. CDr10b presents several major advances to existing labeling methods: Firstly, its ease of use, rapidity and non-toxicity make it superior to existing dyes that rely on longer incubation times [12] or the more technically challenging and time-consuming method of oral gavage [15,16]. Secondly, CDr10b specifically labels the mid-intestine, unlike other vital dyes such as Acridine Orange and Neutral Red that also label necrotic/apoptotic cells and macrophages, respectively [13,24,25]. Thirdly, CDr10b can be used as an in vivo marker of the mid-intestinal segment, including a functional readout of absorptive function that allows the rapid detection of developmental intestinal patterning defects. This work adds a powerful new tool to the zebrafish model and strengthens its use as an in vivo model to study intestinal biology, injury, and repair.

## Supplementary Materials

The following are available online. Figure S1: CDr10b and Neutral Red co-localize in the mid-intestine, Figure S2: CDr10b on sections, Figure S3: Chemical structures and properties of CDr10b and CDr10a, Figure S4: CDr10b as marker for intestinal integrity, Table S1: Summary of staging, duration, dosage and toxicity of CDr10b treatment in larval zebrafish, Table S2: Detailed summary of DEAB-treatment.
