# Supplementary material for: The Vital Dye CDr10b Labels the Zebrafish Mid-Intestine and Lumen
Source: Molecules. 2017 Mar 13;22(3):454. doi: 10.3390/molecules22030454 (PMC6155399; doi:10.3390/molecules22030454)
Supplement: Supplementary file 1 [file molecules-22-00454-s001.pdf]

Supporting Information

The vital dye CDr10b labels the zebrafish mid-intestine and lumen

Veronika Sander [1], Shantanu Patke [1] , Jung Y. Lee[2], Young-Tae Chang[2] and Alan J. Davidson[1,*]

Supplementary Figures and Tables

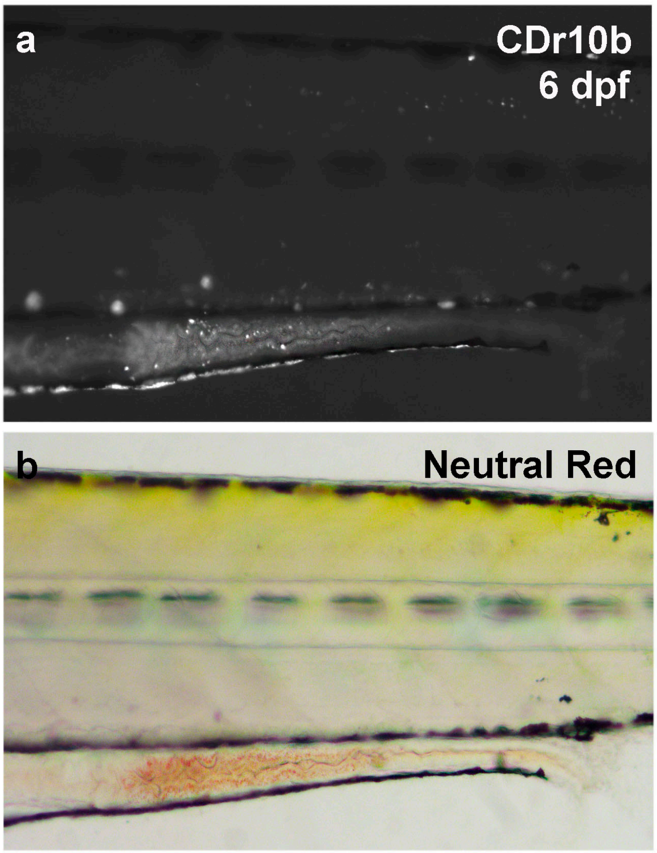

Figure S1. CDr10b and Neutral Red co-localize in the mid-intestine. a) Larva stained with 100 nM CDr10b for 45 min at 5 dpf. b) Same larva stained with 2.5 µg / ml Neutral Red for 5 hrs at 6 dpf. dpf, days post fertilization. Neutral Red staining was performed as described in Oehlers *et al* [13].

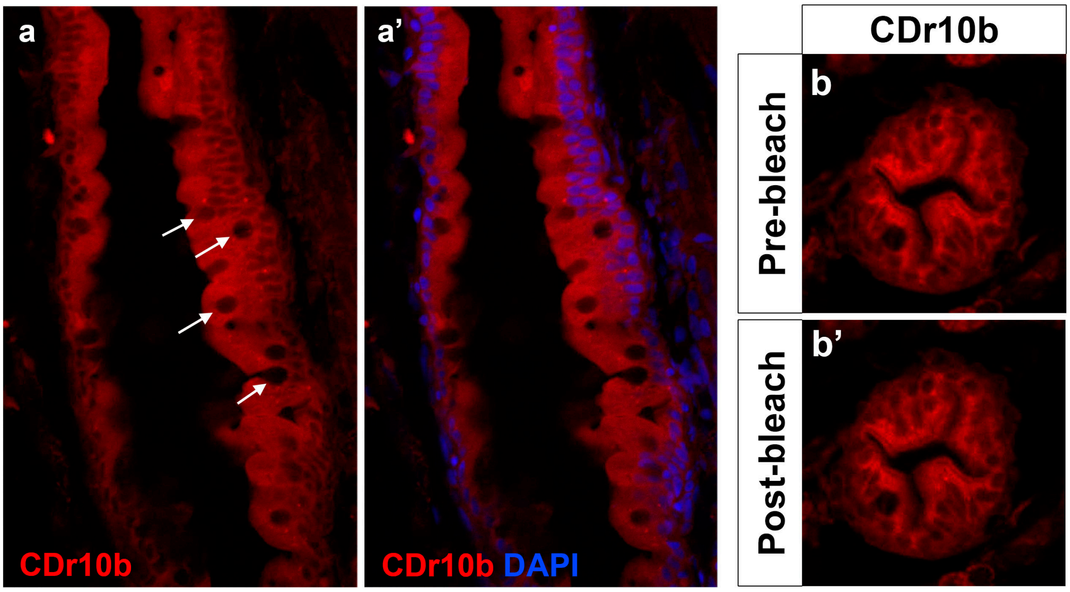

Figure S2. CDr10b on sections. a, a′) Sagittal section through the mid-intestinal segment of a 7 dpf larva shows cytoplasmic CDr10b signal present in all cells, while the nuclei (DAPI, blue) are negative. The dark spots correspond to mucus droplets of goblet cells (arrows). b) Transverse sections before photobleaching. b′) Same sections photographed after 5 min photobleaching, using the same exposure settings.

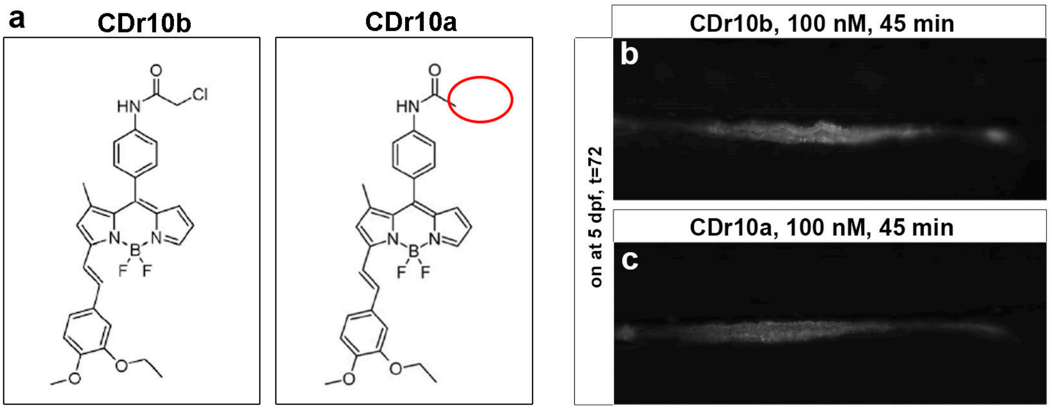

Figure S3. a) Chemical structures and properties of CDr10b and CDr10a. The chloroacetyl motif is absent in CDr10a (red circle). b, c) Intestine-labeling properties of CDr10a, the non-chloroacetylated version of CDr10b. Larvae were treated at 5 dpf with 100 nM CDr10b (a) or CDr10a (b) for 45 min, followed by double washout, daily medium change and imaging at t=72 hrs. No differences in signal intensity or location were detectable. Photos were captured under the same exposure settings. dpf, days post fertilization; t, time post treatment.

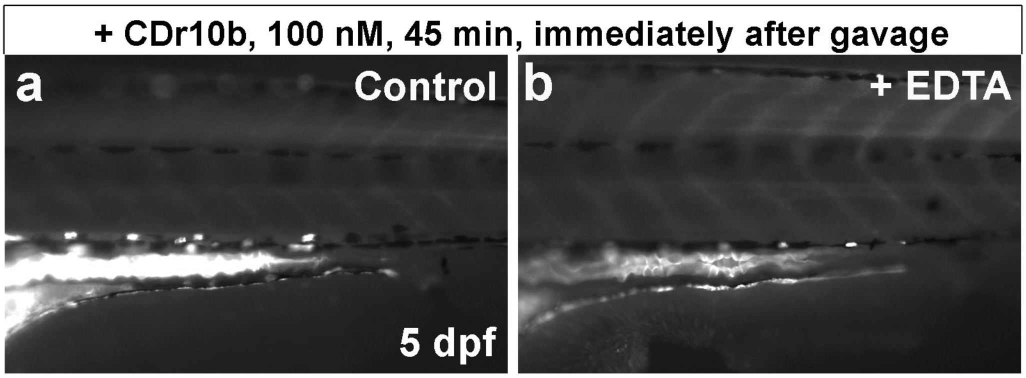

Figure S4. CDr10b as marker for intestinal integrity. a) Control (gavage with E3 larval water) b) Larva after gavage with 20 mM EDTA. EDTA disrupts the tight junctions of the intestinal epithelium releasing CDr10b into surrounding tissues (ventral fin) and the circulation. Note the lower intensity of CDr10b in the leaky (EDTA-damaged) intestinal tube (b) compared to the control (a). The same exposure settings were used for panels a and b. dpf, days post fertilization. Oral gavage was performed as described in Cocchiaro and Rawls [15].

Table S1. Summary of staging, duration, dosage and toxicity of CDr10b treatment in larval zebrafish. n, number; t, time after treatment; dpf, days post fertilization.

CDr10b/a or DMSO on at	Duration	Dose	n	24-hr Survival	Effects at t=24 hrs	Effects at t=48 hrs	Effects at t=72 hrs	Conclusions
CDr10b<br>3 dpf	45 min	100 nM	15	93.3%	CDr10b in yolk and intestinal lumen; cloaca opening ~4 dpf	Little CDr10b in the lumen.	Mid-intestinal cells labeled with CDr10b; small cast formation in ~25% of the larvae	- At 3 – 4 dpf, CDr10b can be used to monitor the progress of intestinal development (opening of the cloaca)<br>- Mid-intestinal cells take up CDr10b from 5 dpf on.<br>- See Figure 3a-c
CDr10b<br>3 dpf	24 hrs	100 nM	19	94.7%	Same as after 45 min but also in circulation			
DMSO<br>3 dpf	24 hrs	0.01%	14	100%	Autofluorescence only	Autofluor. only	Autofluor. only	
CDr10b<br>4 dpf	45 min	100 nM	21	100%	CDr10b in intestinal lumen; excretion by peristalsis; no cell staining			
CDr10b<br>4 dpf	24 hrs	100 nM	17	100%	Same as after 45 min but brighter			
DMSO<br>4 dpf	24 hrs	0.01%	21	90.5%	Autofluorescence only			
CDr10b<br>5 dpf	45 min	100 nM	83	98.8%	Little CDr10b left in the lumen left; mid-intestinal cells specifically labeled with CDr10b		**Effects at t=96 hrs**<br>Survival: 100%, n=16<br>DMSO: 100%, n=10<br>Signal intensity see Figure 3d.	Best dose and duration of treatment for CDr10b readout:<br>100 nM, 45 min , at 5 dpf (Figure 2)
CDr10b<br>5 dpf	24 hrs	100 nM	50	100%	Same as after 45 min but brighter			
DMSO<br>5 dpf	24 hrs	0.01%	70	100%	Autofluorescence only			
CDr10b<br>5 dpf	10 min	100 nM	20	100%	Barely detectable			
CDr10b<br>5 dpf	20 min	100 nM	15	100%	Much weaker than after 45 min			
CDr10b<br>5 dpf	45 min	10 nM	16	100%	Much weaker than with 100 nM			
CDr10b<br>5 dpf	45 min	1 nM	15	93.3%	Barely detectable			
CDr10b<br>5 dpf	45 min	100 pM	22	100%	Not detectable			
CDr10b<br>5 dpf	45 min	100 µM	16	100%	Very bright CDr10b staining in mid-intestinal cells			
CDr10b<br>5 dpf	45 min	100 nM	22	100%				No difference in signal intensity<br>See Figure S2
CDr10a<br>5 dpf	45 min	100 nM	33	100%				

Table S2. Detailed summary of DEAB-treatment. dpf, days post fertilization; n, number; som: somite.

Reagent	Stage	Dose	CDr10b on at	Dose, Duration	n	Phenotypes			Other observations
						Normal	Minor shift to anterior	Major shift to anterior	
DMSO	8-som – 10-som	0.01%	5 dpf	100 nM, 45 min	11	11	0	0	- Minor necrosis<br>- 20% of the larvae are slightly delayed in development (swim bladder not formed by 6 dpf)
DEAB	8-som – 10-som	1 µM	5 dpf	100 nM, 45 min	11	1	8	2	
DMSO	8-som – 15-som	0.01%	5 dpf	100 nM, 45 min	12	12	0	0	- Minor necrosis in DMSO and DEAB-treated larvae<br>- 25 - 50% of the larvae are slightly delayed in development<br>- 20% show small cardiac edema
DEAB	8-som – 15-som	1 µM	5 dpf	100 nM, 45 min	17	0	3	14	
